# Swelling of latex particles—towards a solution of the riddle

**DOI:** 10.1007/s00396-016-3988-2

**Published:** 2016-12-08

**Authors:** Amit Tripathi, Chunxiang Wei, Klaus Tauer

**Affiliations:** Max Planck Institute of Colloids and Interfaces, D-14424 Potsdam, Germany

**Keywords:** Emulsion polymerization, Monomer diffusion, Particle swelling

## Abstract

The assumption that during emulsion polymerization, the monomer molecules simply diffuse through the aqueous phase into the latex particles is a commonplace. However, there are experimental hints that this might not be that easy. Here, simulation results are discussed based on Fick’s diffusion laws regarding the swelling of latex particles. The results of quantitative application of these laws for swelling of latex particles allow the conclusion that the instantaneous replenishment of the consumed monomer during emulsion polymerization requires a close contact between the monomer and the polymer particles.

## Introduction

Starting an aqueous heterophase polymerization outside the monomer drops is the typical scenario of classical emulsion polymerization (EP). This polymerization technique is industrially used since many decades [1, 2], and the kinetics of the process is the topic of numerous scientific papers and textbooks since the middle of the 1940s [3–11].

A key assumption of the widespread and mostly accepted mechanism of EP is the immediate substitution of the monomer consumed by propagation inside the polymer particles by a fresh monomer via diffusion through the aqueous phase as long as monomer droplets (or a free monomer phase) exist^1^ [3, 12]. Accordingly, the monomer concentration inside the latex particles is supposed to be constant until the monomer droplets (the free monomer phase) disappear. This presumption is long lasting even though experimental data of the monomer concentration inside the latex particles during the course of EP do not support it [13–15]. Remarkably, the corresponding results have been obtained with both water-soluble (potassium peroxodisulfate) [13] and oil-soluble (2,2′-azobis(2-methylpropionitril)) [14, 15] initiators whereby, regardless of the initiator, typical emulsion polymerization kinetics has been observed.

Our purpose in writing this short communication is to draw attention to the fact that despite the many accomplishments of industrial EP and chemical engineering with respect to product development and process understanding, respectively, at least one fundamental question remains to be answered.

Harkins’ idea of monomer diffusion, from the reservoir which can be a bulk or dispersed monomer phase, through the aqueous phase to the main reaction loci—the equilibrium swollen monomer polymer particles—appears to be straightforwardly concluded based on undisputable experimental facts. The decisive aspect here is the extremely high rate of polymerization (monomer consumption) achievable with EP despite the spatial separation of monomer and the main reaction loci^2^ [3].

The instantaneous replenishment of the monomer inside the active particles containing a propagating radical requires that the monomer uptake frequency should correspond to at least the propagation frequency. This requirement can be expressed by Eq. (1) where *C*_M,P_ is the monomer concentration inside the particles, *k*_p_ the propagation rate constant, $\tilde{D}$ is the monomer diffusion coefficient, and *x* the distance inside the particle (*x* = 0 is the center of the spherical particle with radius *r*_0_ and *x* = *r*_0_ the distance from the center to the interface). A relation such as Eq. (1) is known also as Thiele modulus (ϕ_Th_) [16, 17] which is a characteristic number, typically describing the ratio between the reaction and the diffusion rate in catalytic reactions.

1 $$ {k}_p{C}_{M,P}=\frac{\tilde{D}}{x^2} $$

**Fig. 1:**
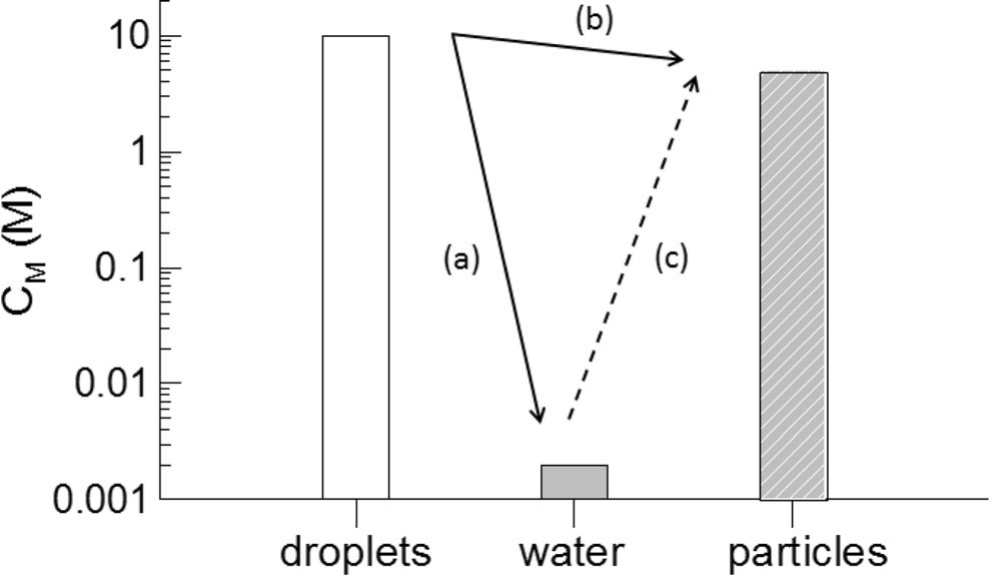
Distribution of the monomer concentration within the components of a batch emulsion polymerization and illustration of possible diffusion paths: **a** from the droplets to the aqueous phase, **b** from the droplets to the particles, and **c** from the aqueous phase to the particles; the monomer concentration at the various loci are illustrated by the values in a typical range which is in the molar range for droplets and particles (in relation to their corresponding volume) but in the millimolar range for the swelling agent or monomer (such as styrene [18, 19]) in water

However, a detailed look at the scenario during aqueous EP reveals a serious problem with this apparently quite logical assumption of an easy monomer diffusion through the aqueous phase (cf. Figure 1). In general, neglecting for the specific moment interactions between components of the reaction mixture, diffusion is the transport of matter from a more concentrated region to a less concentrated region with the aim to equilibrate the chemical potential, here that of the monomer inside the reaction system. Hereinafter, the reaction system comprises only droplets, particles, and water but neglects the gas phase. Figure 1 sketches the situation with respect to the monomer concentration across the EP space and illustrates the problem to be addressed.

## Computation methods, technical information

Fick’s diffusion law for spherical geometry, cf. Eq. (7) below, can be represented in a dimensionless form using the following substitutions:

2 $$ {C}^{*}=C/{C}_0;\kern0.5em {x}^{*}=x/{x}_0;\kern0.5em {D}^{*}=D/{D}_0;\kern0.5em {t}^{*}={D}_0t/{x}_0^2 $$

where *D*_0_ is the diffusion coefficient of the swelling agent at *r* = *r*_0_. This treatment is similar to the approach by Hsu [20]. Using substitutions given above (2), Eqs. (7)–(9) can be expressed as

3 $$ \frac{\partial {C}^{*}}{\partial {t}^{*}}=\frac{\partial }{\partial {x}^{*}}\left({D}^{*}\frac{\partial {C}^{*}}{\partial {x}^{*}}\right)+\frac{2}{x^{*}}\left({D}^{*}\frac{\partial {C}^{*}}{\partial {x}^{*}}\right) $$

4 $$ \begin{array}{llll}\frac{\partial {C}^{*}}{\partial {x}^{*}}=0\hfill & at\hfill & {x}^{*}=0,\hfill & {t}^{*}\ge 0\hfill \end{array} $$

5 $$ \begin{array}{llll}{C}^{\ast }=1\kern.2em & at\kern.2em & {x}^{\ast }=1\kern.3em & {t}^{\ast }>0\end{array} $$

Equation (3) was solved numerically using a finite-difference method similar to [20]. In this approach, the polymer particle is assumed as made of *n* (herein, *n* = 200) spherical shells and the concentration in each shell is calculated by numerical methods. The integration with respect to time (or dimensionless time t*) was done using Matlab r2015a. It should be pointed out that the diffusion coefficient in a polymer is highly dependent on the difference between the actual temperature and the glass transition temperature of the polymer particle changing along with the degree of swelling which suggests that the diffusion coefficient in each shell can be different. The diffusion coefficient of the swelling agent was estimated using the approach suggested by Karlsson et al. [21]. It should be pointed out, that along with the swelling agent water can also hydroplasticize the polymer particle and influence diffusion [22], however, depending on the hydrophilicity of the polymer in different extent.

## Results and discussion

The monomer concentrations at the various spots of EP as sketched in Fig. 1 suggest that a simple concentration gradient-driven diffusion from the monomer drops to the aqueous phase along path (a) is easily possible but that it is rather unlikely along path (c) which is from the aqueous phase directly into the particles. This conclusion is buttressed by estimating the diffusion rates using Fick’s second diffusion law, Eq. (6).

6 $$ \frac{\partial {C}_M}{\partial t}=\tilde{D}\frac{\partial^2{C}_M}{\partial {x}^2} $$

Equation (6) was adapted for spherical geometry according to the treatment of Crank [23] by Eq. (7). This equation was solved to characterize the model-related Eqs. (2–5) diffusion of the monomer (or in general of any swelling agent)^3^ in a spherical unswollen polymer particle of radius *r*_0_.

7 $$ \frac{\partial {C}_M}{\partial t}=\frac{1}{x^2}\frac{\partial }{\partial x}\left({x}^2\tilde{D}\frac{\partial {C}_M}{\partial x}\right) $$

For the estimations, only radial diffusion was considered, and the volume change in the particle was assumed negligible. The total radial change in the particle size for monomer concentration ≤5 M is at maximum about 26%. Note the impact of the particle size change which is anisotropic with respect to the radial distance will be investigated later. The boundary conditions were chosen according to Eqs. (8) and (9).

8 $$ \begin{array}{lllll}\frac{\partial {C}_M}{\partial x}=0\hfill & at\hfill & x=0\hfill & and\hfill & t>0\hfill \end{array} $$

9 $$ \begin{array}{lllll}{C}_M={C}_{M,0}\hfill & at\hfill & x={r}_0\hfill & and\hfill & t\ge 0\hfill \end{array} $$

*C*_M,0_ is the swelling agent concentration at the particle surface (particle with radius *r*_0_) and is assumed to be in equilibrium at any time with the continuous phase.^4^ It is to emphasize that *C*_M,0_ is a model-related fictive value necessary to establish the required concentration gradient driving the swelling process. During swelling, the conditions particularly with respect to viscosity and hence diffusion coefficient inside the particles are changing. Clearly, the values of both *C*_M_ and $\tilde{D}$ in Eqs. (1), (6), and (7) are interdependent. The change of $\tilde{D}$ with an increasing monomer concentration is considered based on experimental data described in [21]. Accordingly, $\tilde{D}$ can be fitted by an empirical model which comprises four different regions (1 > ϕ_m_ > 0.3, 0.3 > ϕ_m_ > 0.15, 0.15 > ϕ_m_ > 0.1, 0.1 > ϕ_m_ > 0) over a range of about 10 orders of magnitude.

**Fig. 2:**
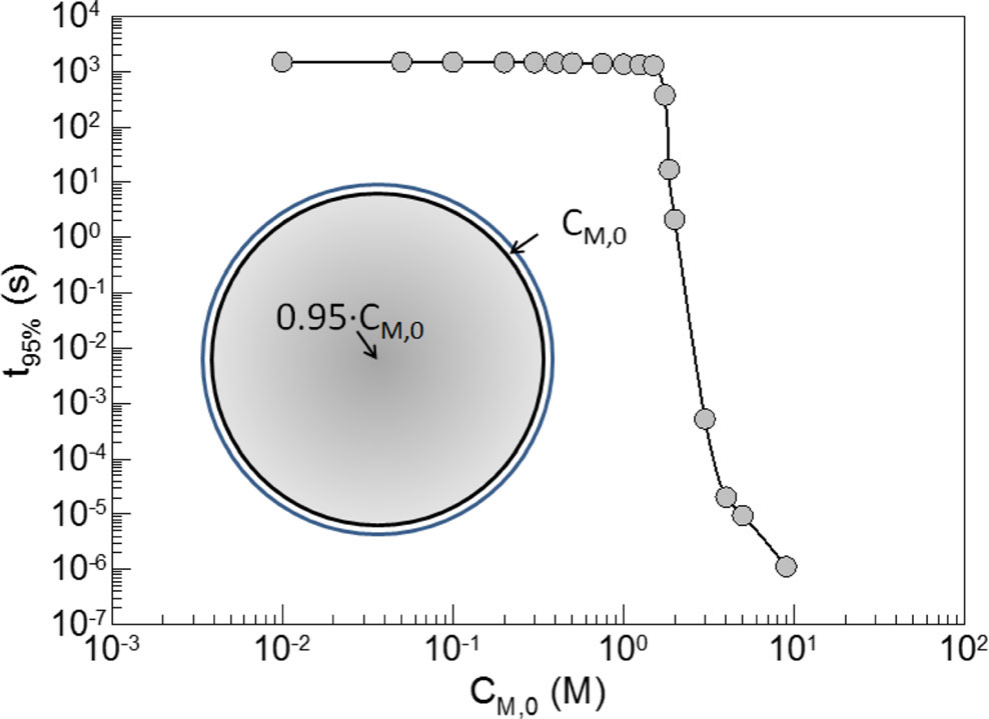
Correlation between the time it takes to swell a particle (diameter 100 nm) to 95% of its equilibrium value (*t*_95%_) and the concentration of the swelling agent in direct contact with particles surface (*C*_M,0_); the *inset sketches* are the assumed scenario when the polymerization was stopped; the *dots* are the simulation results and the *line* is just for guiding the eyes; the swelling agent is supposed to be located with the concentration *C*_M,0_ inside an infinitely thin layer as indicated by the *bright ring* around the particle

Figure 2 shows simulation results for a polymer particle with an unswollen diameter of 100 nm (corresponding to an average dry particle) and a varying concentration of a swelling agent at the interface (*C*_M,0_ as boundary condition). Note, *C*_M,0_ corresponds to the initial concentration difference that thermodynamically drives the swelling process. The time it takes for the swelling agent to penetrate into the particle until the center is saturated to 95% relative to the particular *C*_M,0_—value (*t*_95%_) in dependence on *C*_M,0_ shows in a log—log plot two distinctly different regions. Between 10^−2^ M < *C*_M,0_ ≤ 1.5 M, the time (*t*_95%_) drops only very little (from 4900 and 4150 s) whereas between 1.75 M ≤ *C*_M,0_ < 9 M, it decreases over almost eight orders of magnitude (from 414 to 2.16·10^−6^ s) with increasing *C*_M,0_. Apparently, the range 1.5 M < *C*_M,0_ < 1.75 M is a critical one, because somewhere within this quite narrow range, a value of *C*_M,0_ or the volume fraction (ϕ_M_) exist at which the swelling kinetics changes.

**Fig. 3:**
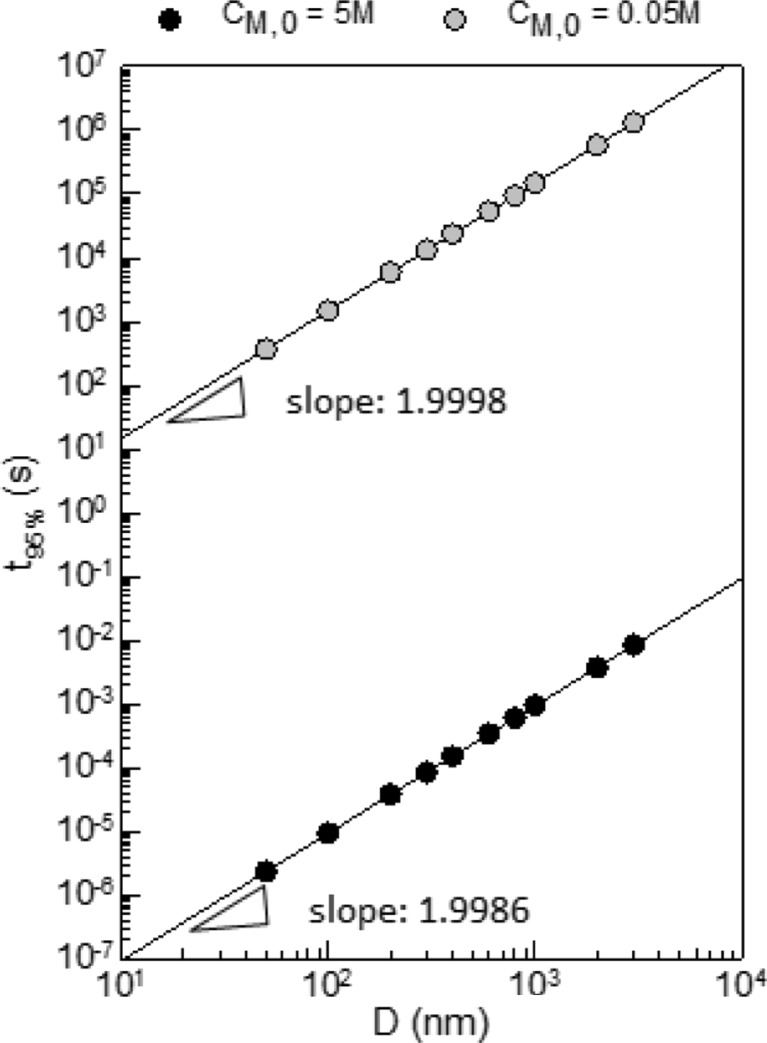
Correlation between the time it takes to swell a particle to 95% of its equilibrium value (*t*_95%_) and the particle diameter (*D*) for two equilibrium concentrations (*C*_M,0_ = 5 and 0.05 M) as indicated

In a typical EP, nonmonodisperse particle size distribution is rather the rule than the exception and hence, the dependence of particles swelling on the average particle size is important. The simulation data put together in Fig. 3 prove the expected quadratic dependence of *t*_95%_ on the particle size exemplarily for only two *C*_M,0_—values above (5 M) and below (0.05 M) the critical range. The overall range of *t*_95%_—values comprises nevertheless quite impressive 14 orders of magnitude.

**Fig. 4:**
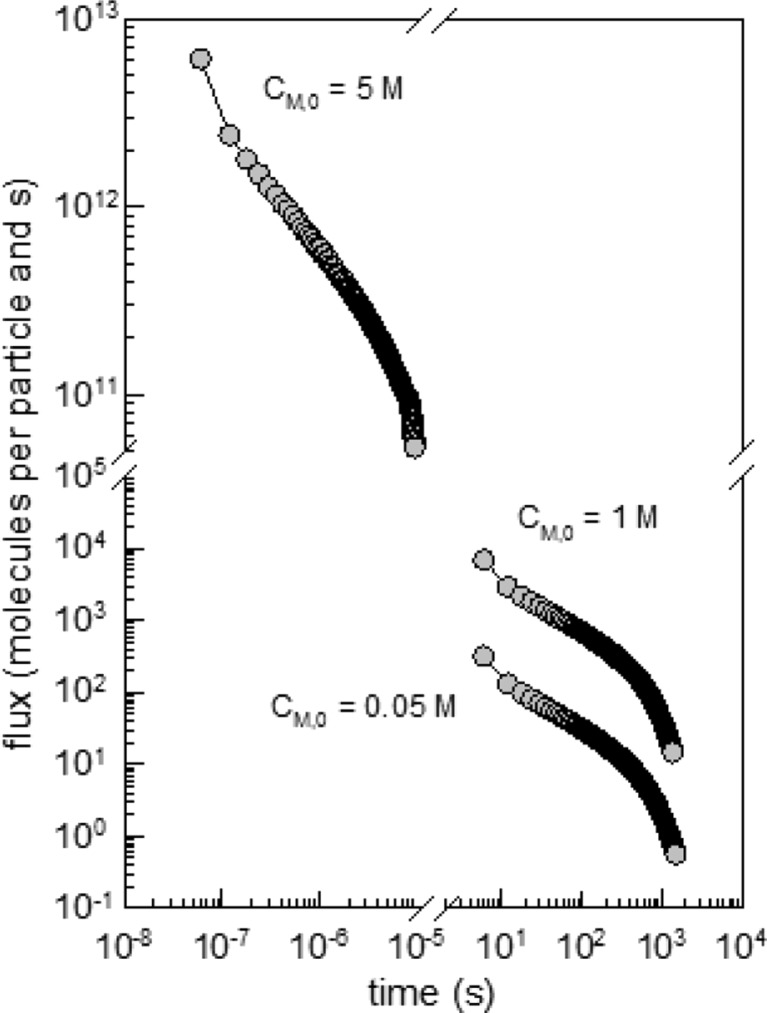
Correlation between the flux of a swelling agent and the swelling time in a latex particle with a diameter of 100 nm for three different equilibrium concentrations as indicated (*C*_M,0_ = 5, 1, and 0.05 M)

The flux of swelling agent (expressed as molecules per particle and seconds) that is needed to swell the particle and to keep *C*_M,0_ constant throughout the whole process is compared in Fig. 4 for three values of *C*_M,0_. The flux of swelling agent stops as soon as it is uniformly distributed across the particle and its concentration equals *C*_M,0_. With an increasing concentration of swelling agent inside the particles (that is with ongoing time), the flux decreases over several orders of magnitude as a consequence of the decreasing driving force (decreasing difference in the chemical potential of the swelling agent with an increasing degree of swelling).

The comprehensive consideration of the simulation results and both the situation given regarding the concentrations as sketched in Fig. 1 together with the experimental facts that EP simultaneously allows high rates of polymerization and the highest molecular weights for free radical polymerization, reveals an apparent riddle with respect to the swelling of latex particles during EP of water-insoluble monomers. The crucial point is to answer the question how does the high monomer concentration, required for both fast monomer diffusion into the latex particles and eventually the high monomer concentration inside, move from the monomer reservoir to the particle interface. To illustrate this, let us consider a single growing radical inside a particle during a styrene emulsion polymerization which consumes *k*_p_ *C*_M,P_ monomer molecules per second. To instantaneously replenish the consumed monomer, it requires an equal amount of monomer molecules diffusing into the particle. The ratio between the consumption of monomer by propagation inside the particle and monomer diffusion into the particle is expressed by the Thiele modulus (ϕ_Th_) (10).

10 $$ {\phi}_{Th}^2=\frac{k_p{C}_{M,P}\cdot {r}_0^2}{\tilde{D}} $$

**Fig. 5:**
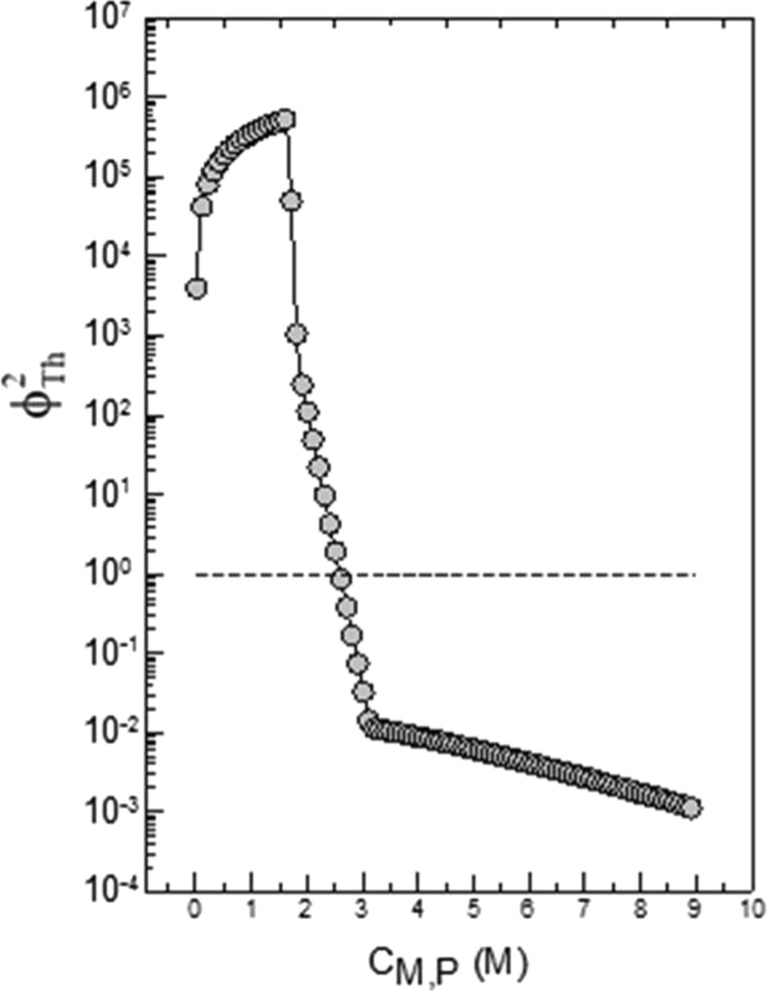
Correlation of the Thiele modulus with the monomer concentration inside a 100-nm particle (*C*_M,P_) containing one polymerizing radical; the calculation was made with Eq. 10 assuming an equilibrium situation with respect to *C*_M,P_ at the particle interface and inside

Figure 5 shows how ϕ_Th_^2^ changes for a single propagating radical in a particle with *r*_0_ = 50 nm in dependence on *C*_M,P_. For this calculation, it is assumed that the particle is equilibrium swollen with the concentration *C*_M,P_ which, according to the equilibrium condition, is equal to *C*_M,0_ at the particle–water interface. For the particular calculation parameters chosen to generate the graph of Fig. 5, the propagation and diffusion frequency are equal (ϕ_Th_^2^ = 1) at *C*_M,P_ of about 2.6 M. For monomer concentration *C*_M,*P*_ ≥ 2.6 M (or ϕ_M_ ≥ 0.26), the monomer diffusion is faster than the propagation, and the equilibrium swelling is maintained. If however, *C*_M,*P*_ < 2.6 M (ϕ_M_ < 0.26) the replenishment of monomer via diffusion is not fast enough and the particle, with respect to monomer, starves out.

In summary, the simulation results using Fick’s second diffusion law with respect to latex particle swelling are clear; they essentially lead to no surprise, and the following conclusions can be drawn. *Firstly*, a high degree of swelling in the molar concentration range as observed for aqueous latex particles (and high monomer concentration during EP) requires a high concentration of swelling agent (monomer during EP) immediately at the particle–water interface. *Secondly*, the concentration of swelling agent (monomer during EP) at the particle interface determines the influx into the particle interior. This means for the situation during EP, that there is a critical monomer concentration above which monomer diffusion is fast enough instantaneously to replenish the consumed monomer. *Thirdly*, as a logical consequence of the simulation results, all situations or measures, that reduce the concentration of the swelling agent (monomer) in an immediate proximity of the particles surface, are of detrimental influence on swelling.

Now let us consider how relevant these conclusions are for better understanding of EP. The second conclusion seems to support the existence of a period during batch EP of constant monomer concentration inside the particles. However, it is to mention that for the estimation of the Thiele modulus (Fig. 5), propagation started in an equilibrium swollen particle which is a special situation and necessarily not given in any EP.

**Fig. 6:**
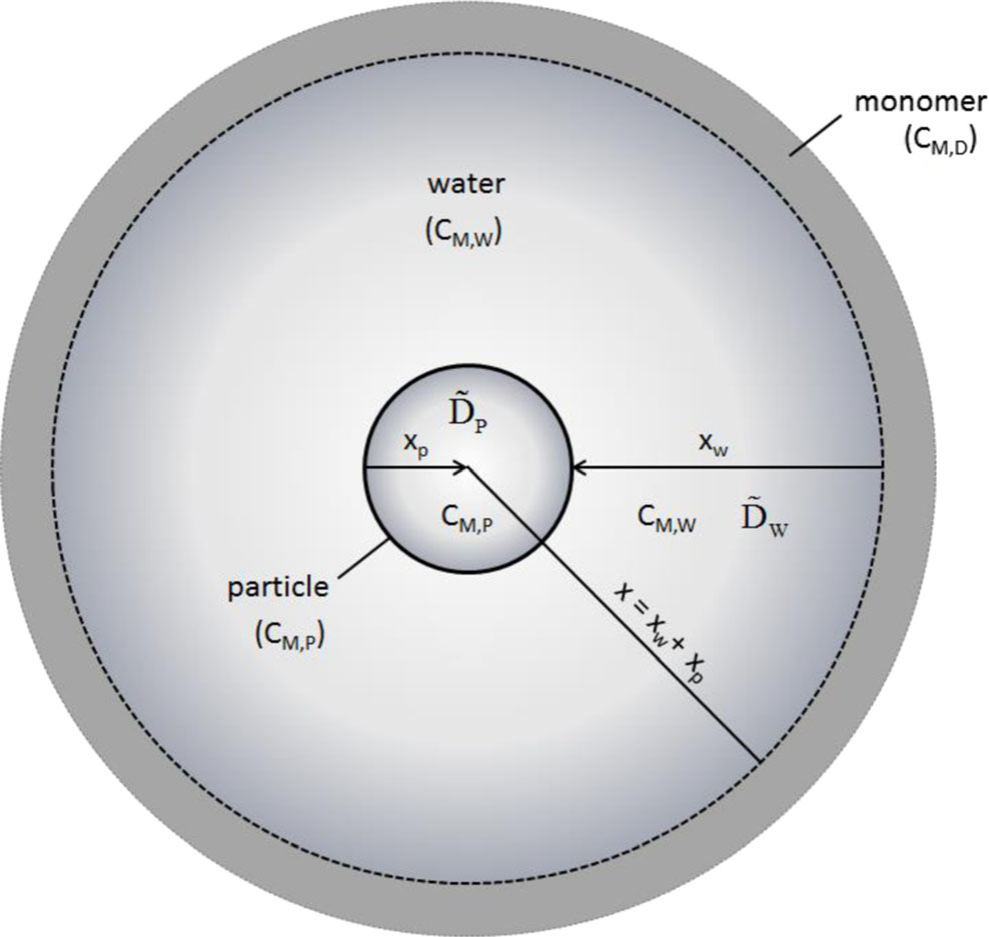
Sketch of the simulation scenario for diffusion of the swelling agent from a source to the particle through the aqueous phase; the particle is placed in the center of a spherical water layer of thickness *x*_w_ which is homogeneously surrounded by a pure swelling agent; for completely penetrating the particle, the swelling agent has to cover the whole distance *x* = *x*_w_ + *x*_p_; the *arrows* indicate the final overall direction of the flux of the swelling agent; *C*_M,D_, *C*_M,W_, and *C*_M,P_ are the concentrations of the monomer in the source, the water, and the particle, respectively; ${\overset{\sim }{D}}_W\kern0.2em and\kern0.2em {\overset{\sim }{D}}_P$ are the diffusion coefficients of the monomer in the water and the particle phase, respectively (note, the latter depends on the fraction of the swelling agent inside the particle)

The implications of the first and third conclusion are much more crucial and universal. The main question is how the required high concentration of hydrophobic monomer with a low solubility in water (cf. Figure 1) is delivered to the water–particle interface, particularly for experimentally observed ϕ_M_—values of about 0.5 (corresponding to a concentration of about 5 M in the latex particles). Necessarily, swelling to such a degree and within a realistic period of time with respect to polymerization requires a correspondingly high concentration in direct contact. The accumulation of a corresponding amount of monomer solely via molecular diffusion through the aqueous phase is not fast enough with respect to time scales relevant to polymerization and hence, it does not contribute to the solution of the riddle. In a certain sense, water as continuous phase acts as quite effective barrier. Within the frame sketched in Fig. 1 for the monomer diffusion, starting from the droplets first into the water and from there into the particles, the following simulation scenario as outlined in Fig. 6 might be helpful to elucidate the issue. Compared to the simulation scenario considered so far, the presence of water as a continuous phase between the monomer drops and the particles increases the complexity. Now, it is necessary to consider both an additional concentration and a diffusion coefficient of the monomer in water as well as the distance between the source (droplet) and the recipient (particle). In order to swell the particle evenly, the monomer has to complete the path first from the droplet–water to the particle–water interface (*x*_w_) and then inside the particle to the center (*x*_p_).

For these calculations, the monomer reservoir was located at a distance of *x*_*w*_ = *z* ⋅ *r*_0_ (z > 1) away from the particle surface. The aqueous phase at this distance, i.e., the droplet–water interface, is assumed to be saturated at all times (*t* ≥ 0) with the swelling agent. At the particle interface (*x*_p_ = *r*_0_) the total swelling agent concentration (*C*_I,M_) is the sum of the concentration on the inner polymer (${C}_{x={r}_{0,P}}$ at *x*_p_ = *r*_0,P_) and outer water side (${C}_{x={r}_{0,W}}$ at *x*_w_ = *r*_0,W_), that is towards the particle’s interior and towards the adjacent aqueous phase, respectively. Also, this interface is assumed to be in equilibrium at all times. This equilibrium is described as a simple distribution coefficient (*K*_d_) which is the ratio of the equilibrium concentration in the particle (*C*_M,P_) and the aqueous phase (*C*_M,W_). In this way, the surface concentration on both sides of the particle can be expressed by Eqs. (11) and (12).^5^

11 $$ {C}_{x={r}_{0,P}}=\frac{K_d}{\left(1+{K}_d\right)}{C}_{I,M} $$

12 $$ {C}_{x={r}_{0,W}}=\frac{1}{\left(1+{K}_d\right)}{C}_{I,M} $$

The time evolution of the concentration at *x* = *r*_0_, that is at the particle surface is estimated by the flux balance given with Eq. (13).

13 $$ \frac{\partial {C}_{I,M}}{\partial t}=-{\tilde{D}}_W\frac{\partial {C}_{x={r}_{0,W}}}{\partial x}+{\tilde{D}}_P\frac{\partial {C}_{x={r}_{0,P}}}{\partial x} $$

As soon as the first monomer molecules reach the particle surface swelling starts. However, the initial rate is lower compared with the case when direct contact between pure swelling agent and polymer was assumed (cf. Figure 2). Due to the slow diffusion inside the particles, monomer accumulates in the interface region of the particles. The particle rapidly swells in an interfacial region, and this highly swollen region expands with an ongoing time towards the center. Obviously, this scenario supports the idea that swelling leads to the formation of an inhomogeneous particle structure as discussed since quite a long time [24–27]. However, complete EP, that is the combination of monomer diffusion into and monomer consumption inside the particles by propagation, is not considered here and results will be reported later.

**Fig. 7:**
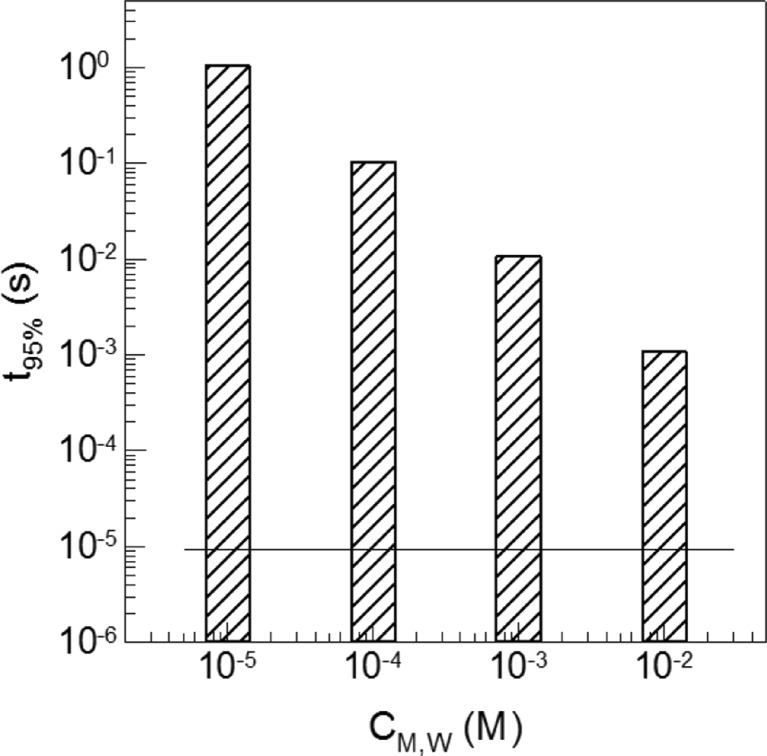
Correlation between the solubility of the monomer in water (*C*_M,W_) and the time to reach particle swelling of 95% (for an equilibrium value of *C*_M,*P*_ = 5 M) according to the scenario as sketched in Fig. 6 for a particle with 100 nm in diameter which is 150 nm away from the monomer surface (*z* = 3); the *line* marks the time which was obtained for such a particle in direct contact with the monomer (cf. Figure 2)

The simulation data compared in Fig. 7 reveal two remarkable details. Firstly, the water phase between the monomer and the particle acts indeed as an effective barrier and drastically increases the time until the equilibrium is reached. Secondly, the data show quite a strong, almost linear influence of the solubility of the monomer in water on the swelling kinetics in the log—log plot. Increasing the water solubility of the monomer by a certain factor also decreases the time to reach the equilibrium swelling almost by the same factor. This result is in qualitative agreement with experimental experience showing that heterophase polymerization of extremely hydrophobic monomers such as lauryl methacrylate needs special measures in order to avoid an excessive formation of coagulum.^6^

The influence of the hydrophilicity of the monomer is much stronger than that of the average distance between the monomer–water interface and the particle surface (*x*_w_). Increasing the distance from 150 nm to 1 μm, this corresponds to a decrease in the overall volume fraction of the colloidal objects by about a factor of 100, only marginally prolongs the time to reach equilibrium from 1.074 to 1.44 milliseconds.

For the simulations, it is easily possible to position *C*_M,0_ at the interface which in reality means that there should be a monomer rich phase between water and the particles. To prove such scenario in experiments with latex particles under conditions relevant to EP is an extremely hard task. Luckily, few model experiments have been described [29–31] supporting the possibility of such layer formation. One set of data proves the accumulation of alkanes at the interface of polystyrene latex particles with ellipsometric light scattering [29]. Other experimental data support the idea that a direct contact between a swelling agent and a polymer is necessary for fast swelling by studies with bulk polymer samples [30, 31]. Very recently, it was shown that the swelling of a bulk polymer samples embedded in water with the swelling agent placed on top does not take place within several hours in the absence of stirring but begins immediately after switching the stirrer on. The importance of the direct contact between drops and polymer for the transfer of matter was evidenced by tinting the polymer with the extremely hydrophobic dye Hostasol Yellow^7^ [31].

There is, however, still another fact which has to be taken into account. This is the thermodynamic force causes to congregate the swelling agent and the particles along the gradient in the chemical potential (μ) [32]. The driving force (F = −dμ/dx) is the entropy maximization or the minimization of the free energy in the system of swelling latex particles. How strong a force this tendency can generate is illustrated by the accumulation of micron-sized latex particles at the quiescent swelling agent–latex interface against the action of gravity [19, 31].

Experimental evidence exists also in supporting the third indirect conclusion drawn from the simulation results regarding a possible hindrance of mass transfer between the monomer layer and the particles [31]. Assuming that the swelling pressure measurements are a way to characterize the swelling process, it was shown that a surfactant layer around the monomer drops can quite effectively hinder the transfer process. The swelling rates of polystyrene with ethylbenzene in stirred systems were the fastest in the absence of surfactants, the second fastest in the presence of a nonionic surfactant, and the slowest in the presence of sodium dodecyl sulfate.

## Conclusion

The simulation studies of the swelling of latex particles based on Fick’s second law of diffusion support the recent experimental findings that the fast swelling of latex particles requires a direct contact of the components. The consequences for aqueous EP are quite significant because stirring supports the fast uptake of monomer by the latex particles due to facilitating contacts between droplets and particles but stabilizer layers delay the process due to hindering the transport across the interface. The simulation results based on Harkins’ idea [3], that in EP, the monomer drops serve “as a storehouse from which the molecules diffuse (through) the aqueous phase ··· into ··· latex particles”, show that the details of this process are crucial and need to be elaborated. Interestingly, the simulation data theoretically back experimental findings showing that the accumulation of monomer at the particle–water interface is crucial for fast swelling.

## Footnotes

1 This idea dates back to 1947 when Harkins in his seminal paper on emulsion polymerization kinetics stated that the role of the monomer drops is “to act as a storehouse of monomer from which its molecules diffuse into the aqueous phase and from this into either soap micelles or polymer monomer latex particles” [3]. Even until today, this idea is repeated in the state-of-the-art textbooks saying that in the “presence of monomer droplets,the monomer-swollen particles grow and the monomer concentration within these particles is kept constant by monomer diffusing through the water phase from the monomer droplets” [12].

2 The high polymerization rate of EP is caused mainly by the spatial separation of a primary radical generation in the continuous phase and radical propagation in the polymer particles where the provided particle size is small enough, the growing radical is isolated, and effectively protected against frequent termination.

3 Henceforth, the term monomer and swelling agent will be used interchangeably.

4 Assuming a molar volume of 100 cm^3^/mol for the swelling agent, a value of *C*_M,0_ = 10 M corresponds to the pure swelling agent at the particle interface or to a volume fraction of the swelling agent ϕ_M_ = 1. Correspondingly, the other *C*_M,0_—values represent smaller ϕ_M_—values which together with the polymer volume fraction (ϕ_P_) add to one.

5
*K*_d_ is used to calculate the development of C_M,P_ with time according to *C*_*M* , *P*_(*t*) = *K*_*d*_ ⋅ *C*_*M* , *W*_(*t*) which in turn is used to estimate the diffusion coefficient.

6 For instance, emulsion polymerization of lauryl methacrylate under a kind of standard conditions with potassium peroxodisulfate as initiator and sodium lauryl sulfate as emulsifier leads to 17% of the polymer in the form of latex and 83% in the form of coagulum. The application of a more hydrophobic initiator leads to a drastically increased latex yield [28].

7 Hostasol Yellow or Solvent Yellow 98 or Fluorescent Yellow 3G is with its chemical name 2-Octadecyl-1H-thioxantheno[2,1,9-def]isoquinoline-1,3(2H)-dione (C_36_H_45_NO_2_S, CAS Registry Number:12671-74-8/27870-92-4) and listed as a water-insoluble dye
